# Supplementary material for: Polymorphisms within the SARS-CoV-2 Human Receptor Genes Associate with Variable Disease Outcomes across Ethnicities
Source: Genes (Basel). 2023 Sep 14;14(9):1798. doi: 10.3390/genes14091798 (PMC10531089; doi:10.3390/genes14091798)
Supplement: Supplementary file 1 [file genes-14-01798-s001.zip › genes-2594619-supplementary.pdf]

Supplementary material:

Supplementary Table S1: Genotype frequencies of *ACE2*, *TMPRSS2*, *NRP1* and *CD147* SNPs in patients with and without clinical presentations across ethnic groups.

Notes: No CP – No clinical presentations, CP – clinical presentations.

Gene/ SNP	Genotype	Black		Caucasian		Indian	
		No CP	CP	No CP	CP	No CP	CP
ACE2 – rs2285666	CC	0.46	0.51	0.75	0.64	0.54	0.32
	CT	0.53	0.41	0.25	0	0.23	0.36
	TT	0.01	0.08	0	0.36	0.23	0.32
TMPRSS – rs12329760	CC	0.22	0.31	0.50	0.65	0.73	0.58
	CT	0.64	0.63	0.50	0.35	0.23	0.37
	TT	0.14	0.06	0	0	0.04	0.05
NRP1– rs10080	AA	0.79	0.68	0	0.15	0.09	0.22
	AG	0.15	0.29	0.40	0.65	0.61	0.49
	GG	0.06	0.03	0.60	0.20	0.30	0.29
CD147 – rs8259	TT	0.20	0.51	0.80	0	0.04	0.13
	TA	0.62	0.41	0.20	0.70	0.44	0.43
	AA	0.18	0.08	0	0.30	0.52	0.44

Supplementary Table S2: A summary of the comparison of COVID-19 disease states (No clinical presentations versus clinical presentations) within each of the four SNPs across African and South Asian individuals

Ethnicity	SNP	No Clinical presentations (Event counts)	Clinical presentations (Event counts)	Genotype comparison	OR (95% CI)	P-value
African	rs2285666	CC (46); TT (1)	CC (92); TT (15)	CC vs TT	7,5 (1,164 to 80,89)	0,024
		CC + CT (99); TT (1)	CC + CT (166); TT (15)	CC + CT vs TT	8,9(1,468 to 95,52)	0,131
		CC (46); CT + TT (54)	CC (92); CT + TT (89)	CC vs CT + TT	0,82(0,5129 to 1,355)	0,4569
		C (145); T (55)	C (258); T (104)	C vs T	1,1 (0,7291 to 1,551)	0,7702
	rs12329760	CC (20); TT (13)	CC (54); TT (11)	CC vs TT	0,3134(0,1222 to 0,8045)	0,024
		CC + CT (78); TT (13)	CC + CT (163); TT (11)	CC + CT vs TT	0,4049 (0,1826 to 0,9571)	0,0419
		CC (20); CT + TT (71)	CC (54); CT + TT (120)	CC vs CT + TT	0,6260 (0,3444 to 1,138)	0,1492
		C (98); T (84)	C (217); T (131)	C vs T	0,7043 (0,4910 to 1,012)	0,0628
	rs10080	AA (79); GG (6)	AA (122); GG (5)	AA vs GG	0,5396 (0,1806 to 1,919)	0,3545
		AA + AG (94); GG (6)	AA + AG (175); GG (5)	AA + AG vs GG	0,4476 (0,1515 to 1,571)	0,2081
		AA (79); AG + GG (21)	AA (122); AG + GG (58)	AA vs AG + GG	1,788 (1,018 to 3,129)	0,0526
		A (173); G (27)	A (177); G (63)	A vs G	2,281 (1,402 to 3,708)	0,0013
	rs8259	TT (20); AA (18)	TT (47); AA (33)	TT vs AA	0,7801 (0,3532 to 1,740)	0,5562
		TT + TA (81); AA (18)	TT + TA (148); AA (33)	TT + TA vs AA	1,003 (0,5229 to 1,876)	>0,9999
		TT (20); TA + AA (79)	TT (47); TA + AA (134)	TT vs TA + AA	0,7218 (0,3963 to 1,319)	0,3077

		T (101); A (97)	T (195); A (167)	T vs A	0,8917 (0,6314 to 1,260)	0,5362
South Asian	rs2285666	CC (12); TT (5)	CC (56); TT (57)	CC vs TT	2,443 (0,7846 to 6,554)	0,1242
		CC + CT (17); TT (5)	CC + CT (121); TT (57)	CC + CT vs TT	1,602 (0,5546 to 4,117)	0,4685
		CC (12); CT + TT (10)	CC (56); CT + TT (122)	CC vs CT + TT	2,614 (1,119 to 6,504)	0,0537
		C (29); T (15)	C (177); T (179)	C vs T	1,955 (1,036 to 3,646)	0,0544
	rs12329760	CC (16); TT (1)	CC (104); TT (10)	CC vs TT	1,538(0,2196 to 17,69)	>0,9999
		CC + CT (21); TT (1)	CC + CT (170); TT (10)	CC + CT vs TT	1,235(0,1872 to 14,03)	>0,9999
		CC (16); CT + TT (6)	CC (104); CT + TT (76)	CC vs CT + TT	1,949 (0,7238 to 5,082)	0,2503
		C (37); T (7)	C (274); T (86)	C vs T	1,659 (0,7306 to 4,023)	0,262
	rs10080	AA (2); GG (7)	AA (40); GG (52)	AA vs GG	0,3714 (0,07537 to 1,866)	0,2986
		AA + AG (16); GG (7)	AA + AG (129); GG (52)	AA + AG vs GG	0,9214 (0,3578 to 2,354)	0,8126
		AA (2); AG + GG (21)	AA (40); AG + GG (142)	AA vs AG + GG	0,3381 (0,07586 to 1,404)	0,1757
		A (18); G (28)	A (169); G (193)	A vs G	0,7342 (0,3999 to 1,399)	0,3506
	rs8259	TT (1); AA (12)	TT (24); AA (79)	TT vs AA	0,2743 (0,02464 to 1,706)	0,2931
		TT + TA (11); AA (12)	TT + TA (101); AA (79)	TT + TA vs AA	0,7170 (0,3121 to 1,641)	0,5081
		TT (1); TA + AA (22)	TT (24); TA + AA (156)	TT vs TA + AA	0,2955 (0,02740 to 1,843)	0,3207
		T (12); A (34)	T (125); A (235)	T vs A	0,6635 (0,3449 to 1,298)	0,3203

Supplementary Figure S1:

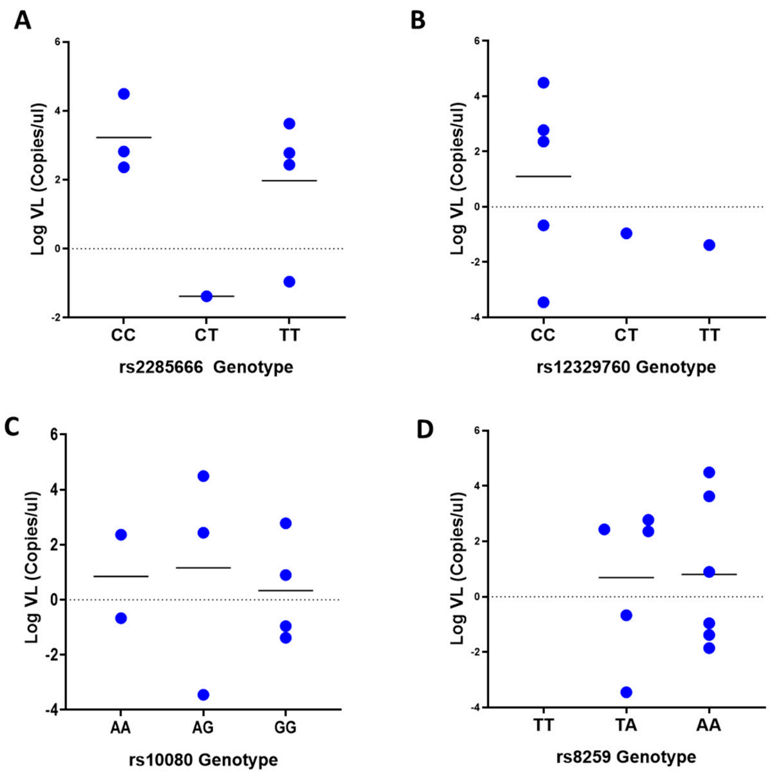

Supplementary Figure S1: SARS-CoV-2 viral load and Genotypes in South Asian individuals.

Supplementary Figure S2:

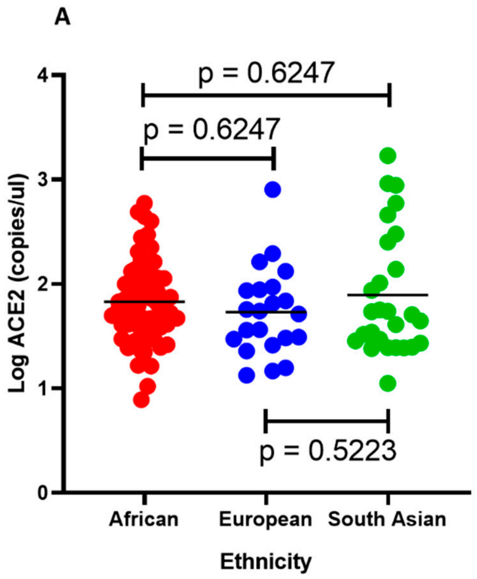

Supplementary Figure S2: The expression of (A) *ACE2* and (B) *NRP1* across ethnic groups in South Africa

Supplementary Figure S3:

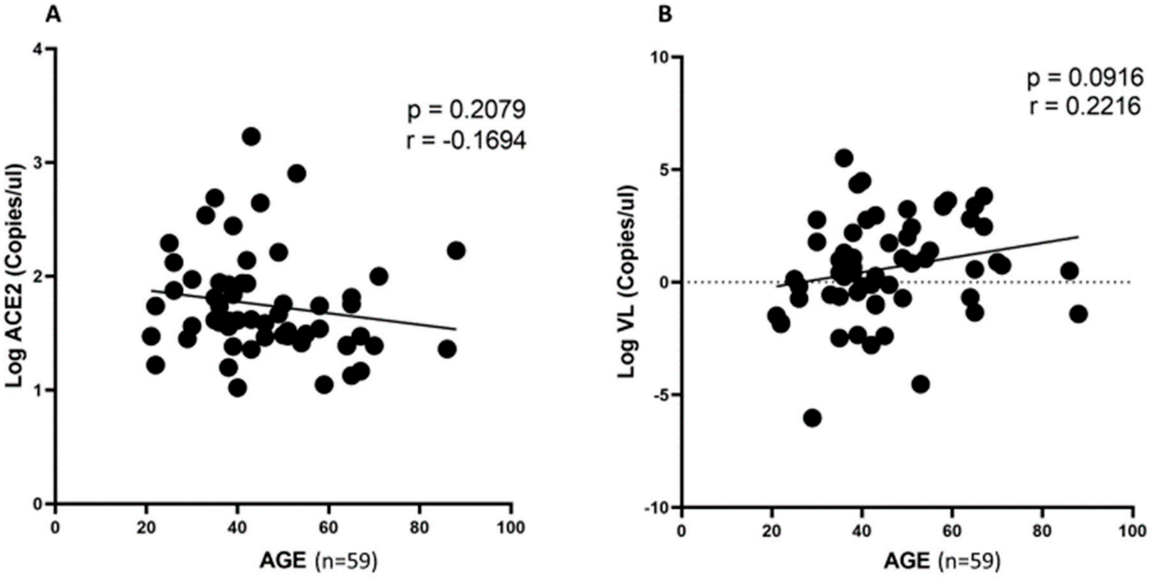

Supplementary Figure S3: Linear regression comparison of ACE2 expression and SARS-CoV-2 viral load against age. (A) Nasopharyngeal *ACE2* expression negatively correlates with age (p = 0,2079), while (B) SARS-CoV-2 viral load positively correlates with age (p = 0,0916). However, both comparisons are not significant.
